# Sunlight exposure and cardiovascular risk factors in the REGARDS study: a cross-sectional split-sample analysis

**DOI:** 10.1186/1471-2377-14-133

**Published:** 2014-06-19

**Authors:** Shia T Kent, Mary Cushman, George Howard, Suzanne E Judd, William L Crosson, Mohammad Z Al-Hamdan, Leslie A McClure

**Affiliations:** 1 Department of Epidemiology, 1665 University Blvd, University of Alabama at Birmingham, Birmingham 35294, Alabama; 2 Department of Medicine, University of Vermont College of Medicine, Burlington VT 05405, Canada; 3 Department of Biostatistics, 1665 University Blvd, University of Alabama at Birmingham, Birmingham 35294, Alabama; 4 National Space Science and Technology Center, NASA Marshall Space Flight Center, 320 Sparkman Drive, Huntsville 35805, Alabama

**Keywords:** Sunlight, Temperature, Weather, Climate, Environment, Blood pressure, Lipids and lipoproteins

## Abstract

**Background:**

Previous research has suggested that vitamin D and sunlight are related to cardiovascular outcomes, but associations between sunlight and risk factors have not been investigated. We examined whether increased sunlight exposure was related to improved cardiovascular risk factor status.

**Methods:**

Residential histories merged with satellite, ground monitor, and model reanalysis data were used to determine previous-year sunlight radiation exposure for 17,773 black and white participants aged 45+ from the US. Exploratory and confirmatory analyses were performed by randomly dividing the sample into halves. Logistic regression models were used to examine relationships with cardiovascular risk factors.

**Results:**

The lowest, compared to the highest quartile of insolation exposure was associated with lower high-density lipoprotein levels in adjusted exploratory (−2.7 mg/dL [95% confidence interval: −4.2, −1.2]) and confirmatory (−1.5 mg/dL [95% confidence interval: −3.0, −0.1]) models. The lowest, compared to the highest quartile of insolation exposure was associated with higher systolic blood pressure levels in unadjusted exploratory and confirmatory, as well as the adjusted exploratory model (2.3 mmHg [95% confidence interval: 0.8, 3.8]), but not the adjusted confirmatory model (1.6 mg/dL [95% confidence interval: −0.5, 3.7]).

**Conclusions:**

The results of this study suggest that lower long-term sunlight exposure has an association with lower high-density lipoprotein levels. However, all associations were weak, thus it is not known if insolation may affect cardiovascular outcomes through these risk factors.

## Background

Cardiovascular health varies with season, weather, and climate [1-3]. While seasonal temperature variation has been a primary target of investigation, sunlight also varies seasonally and has not been adequately investigated. Sunlight directly alters vitamin D status, but aside from skin cancer there are few data on how sunlight directly affects human health [4-6]. Although there are few studies of vitamin D and stroke, there is indication that vitamin D insufficiency may increase vascular event risk factors [4,5,7]. Both geographic latitude and vitamin D level have been linked to blood pressure, with potential mechanisms involving the renin-angiotensin system, inflammation, vasculature, or glycemic control [8,9]. Exposure to ultraviolet B radiation has also been shown to affect blood pressure and other stroke risk factors [7,8,10]. Vitamin D and cholesterol have a common upstream metabolite 7-dehydrocholesterol, which is converted to previtamin D_3_ in the skin after exposure to sunlight [11]. Observational studies have shown that higher vitamin D blood levels may improve lipid levels [12]. Higher vitamin D levels may also improve health status of those with chronic kidney disease, although the results are mixed [13]. Inflammation is related to stroke, blood pressure, lipid levels, and kidney function, and may also be related to vitamin D levels [14-16]. There are seasonal variations in inflammation, although this could be due to infection and allergy [16,17]. Vitamin D may also improve kidney function by acting as renin-angiotensin system inhibitors [18] and improving microalbuminuria [19].

Sunlight radiation and temperature are available from the North American Land Data Assimilation System Phase 2 (NLDAS-2) forcing. These data were matched to an individual’s geocoded home residence and have previously been used in the REasons for Geographic And Racial Differences in Stroke (REGARDS) study, finding that reduced sunlight exposure was associated with increased stroke incidence [20]. In this manuscript, we examine whether increased residential sunlight exposure is related to increased blood pressure, serum lipid levels, kidney function, and inflammation. Since both skin color and the kidney are linked with vitamin D production and regulation [21,22], and since Vitamin D levels have been posited to contribute to racial health disparities [23], we examine whether increased sunlight radiation exposure leads to poorer outcomes among black participants and those with impaired kidney function. To account for the multiple hypotheses we are testing, we perform a split-sample replication analysis. The large size of the REGARDS cohort allows us to split the participants into two samples: a hypothesis-generating sample to explore possible significant relationships, and a confirmatory sample to independently assess the associations found in the exploratory analyses, reducing the likelihood of Type I error. We hypothesize that increased sunlight exposure is related to improved cardiovascular risk factor status in both exploratory and confirmatory samples of the REGARDS study.

## Methods

### Study participants

REGARDS is a longitudinal study of United States (US) participants aged 45 years and older [24]. The REGARDS study was designed to investigate reasons underlying the higher rate of stroke mortality among blacks, compared with non-Hispanic whites, and among residents in the Southeastern United States, compared with other US regions. At baseline, 56% of the participants were residing in the stroke belt (an area of the US with high stroke mortality in the Southeast including GA, NC, SC, AL, TN, LA, AR, MS), with the remaining 44% from the rest of the contiguous 48 US. Participants from the stroke buckle (an even higher stroke mortality region comprised of the coastal plains of NC, SC, and GA) comprised 21% of the REGARDS population. The cohort population at baseline was 42% African-American/58% white and 45% male/55% female. Further details on the study are available elsewhere [24]. The REGARDS study and the current analysis were approved by the Institutional Review Boards of participating institutions.

### Data collection

At baseline, a telephone interview was conducted during which informed consent was obtained and the participant’s self-reported demographic and behavioral factors, and medical history were recorded. Participants were then visited in their homes by a trained health professional who collected blood pressure, height, weight, blood, urine, and conducted electrocardiograms (ECGs), and obtained written informed consent. Blood and urine were sent to a central repository at the University of Vermont and ECG data were read at Wake Forest University. The examiner also left a residential history form to fill and mail.

### Assessment of sunlight exposure and temperature

We used data from the National Aeronautics and Space Administration (NASA) – National Oceanic and Atmospheric Administration (NOAA) NLDAS-2 dataset to determine sunlight radiation and temperature. The NLDAS-2 dataset is based on model reanalysis data and remotely-sensed and ground observations, and consists of a grid surface with ~14 km resolution over North America [25]. NLDAS-2 solar radiation that was assessed at one-hour intervals was used to calculate a daily total referred to herein as daily “insolation” [26]. For this study, we merged daily insolation and maximum air temperatures with data from REGARDS’ residential history form, which consists of locations where the participant had lived prior to enrollment into REGARDS, along with age when relocating. Each location the participant recorded was matched to a feature in the US Geological Survey’s Geographic Names Information System using ArcGIS 9.3. For participants who had a period of missing residential data, due to having an unidentifiable location or residence outside of the contiguous 48 United States, we used only the existing residential history to compute environmental exposure averages. We assumed participants moved during July of the indicated moving year.

As in our previous studies, we calculated each month’s average daily insolation and temperature exposure at each participant’s residential location to estimate each participant’s average exposure for the year previous to baseline [20,27]. We then categorized insolation and temperature exposure into quartiles. In order to capture extreme exposures, we also categorized insolation and temperature exposure using cutpoints at the 5^th^ and 95^th^ percentiles.

### Outcomes

Blood pressure was measured during the REGARDS in-home visit by a trained technician using a standard protocol and regularly tested aneroid sphygmomanometer and was calculated as an average of two measurements taken after the participant was seated for five minutes. Hypertension was defined as present with self-reported use of antihypertensive medications, systolic blood pressure (SBP) ≥ 140 mm Hg, or a diastolic blood pressure (DBP) ≥ 90 mm Hg. Blood was collected during the in-home visit, and shipped to the central laboratory at the University of Vermont using standard protocols. Standard assays were used to determine lipid levels and high-sensitivity C-reactive protein (CRP) assays were used to determine the level of CRP, which was log transformed due to a skewed distribution. CRP levels were categorized into low/medium risk (CRP ≤ 3 mg/dL) and high risk (CRP > 3 mg/dL). Dyslipidemia was defined as present with self-reported use of lipid-lowering medication, total cholesterol ≥ 240 mg/dL, low-density lipoprotein (LDL) ≥ 160 mg/dL, or high-density lipoprotein (HDL) ≤ 40 mg/dL. Kidney function was determined by the estimated glomerular filtration rate (eGFR) computed using the CKD EPI equation (participants with eGFR < 60 classified as having impaired kidney function) [28].

### Statistical methods

**Figure 1:**
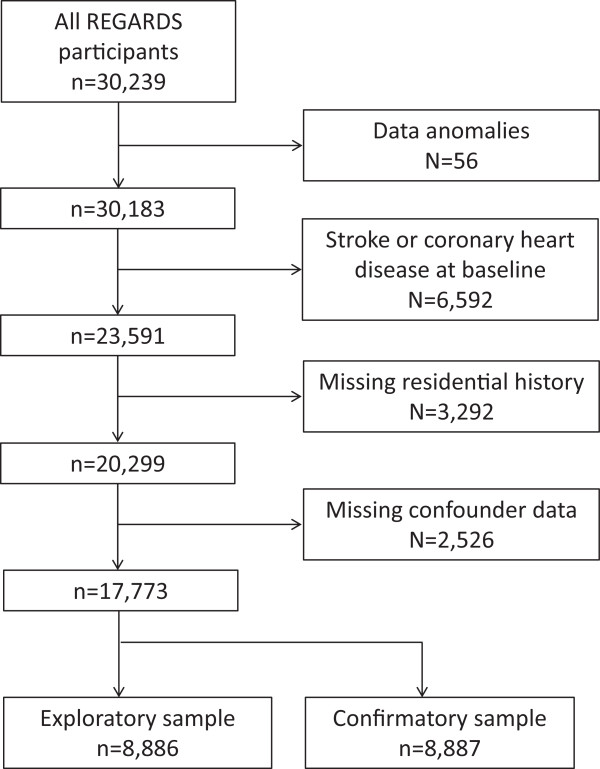
Eligibility flowchart for participants included in the analysis of sunlight and cardiovascular risk factors.

Because some confounders had missing data for large numbers of participants, we attempted to minimize selection bias by creating a separate “missing” category for any variable that had >1,000 participants missing data. Participants were excluded due to data anomalies, stroke or coronary heart disease at baseline, missing residential history, and missing confounder data (for those will <1,000 participants missing data). Of the 30,239 participants enrolled at baseline, 17,773 participants were available for analyses (Figure 1).

To perform a split-sample replication analysis we randomly assigned the eligible participants into one of two samples of equal size. In the first “exploratory” sample, we ran multivariable logistic or linear regression models adjusting for temperature, age, race, region (stroke belt, stroke buckle, or non-stroke belt), gender, education (less than high school, high school graduate, some college, or college graduate), income (<$20,000, $20,000 to $34,999, $35,000 to $74,999, or ≥ $75,000), quartiles of vitamin D intake, exercise (none, 1 to 3 times/week, or ≥ 3 times/week), alcohol use (none, moderate: ≤ 1 drink per day for women or ≤ 2 drinks per day for men, or heavy: > 1 drink per day for women and > 2 drinks per day for men), smoking status (current, past, or never) and body mass index (<18.5, 18.5 to 24.9, 25 to 29.9, or ≥ 30). We also adjusted for statin use in models with cholesterol, HDL, or LDL as the outcome, and adjusted for antihypertensive medication use in the models with SBP as the outcome. Using interaction terms in fully adjusted models, we tested for multiplicative interactions between insolation and each of race or impaired kidney function on our outcome variables. In the second “confirmatory” sample, we re-ran all models for outcomes with significant associations in the explanatory sample.

This study was approved by Institutional Review Boards of participating institutions.

## Results

### Exploratory and confirmatory samples

The exploratory (n = 8886) and confirmatory (n = 8887) samples did not significantly differ by most variables (Table 1; Additional file 1: Table S1). The exception is that the confirmatory sample had a higher proportion of males, although the difference in proportions was only 2.1% (p = 0.0041).

**Table 1:** Meteorological and secondary stroke endpoint distributions by insolation exposure in the exploration (n = 8886) and confirmatory (n = 8887) samples

**Characteristic**	**Exploratory sample**	**Confirmatory sample**	**P-value**
Quartiles of insolation, n (%)			
1st (12327 to 15565 KJ/m2/day)	2207 (24.8%)	2236 (25.2%)	
2nd (15565 to 16724 KJ/m2/day)	2180 (24.5%)	2263 (25.5%)	0.22
3rd (16724 to 17724 KJ/m2/day)	2223 (25.0%)	2220 (25.0%)	
4th (17724 to 22733 KJ/m2/day)	2276 (25.6%)	2168 (24.4%)	
Maximum Temperature, n (%)			
1st quartile (8.5 to 17.8°C)	2206 (24.8%)	2237 (25.2%)	
2nd quartile (17.8 to 21.7°C)	2247 (25.3%)	2198 (24.7%)	0.82
3rd quartile (21.7 to 24.0°C)	2224 (25.0%)	2217 (24.9%)	
4th quartile (24.0 to 31.7°C)	2209 (24.9%)	2235 (25.1%)	
SBP, mean mmHg (SD)	126.5 (16.4)	126.4 (16.0)	0.49
Cholesterol, mean mg/dL (SD)	195.0 (38.2)	194.3 (37.8)	0.25
LDL, mean mg/dL (SD)	116.8 (34.0)	116.3 (33.9)	0.34
HDL, mean mg/dL (SD)	53.3 (16.3)	53.2 (16.1)	0.24
Ln CRP, mean mg/dL (SD)	0.77 (1.16)	0.75 (1.18)	0.17
Hypertensive, n (%)	4847 (54.5%)	4760 (53.6%)	0.19
Dyslipidemia, n (%)	4778 (53.8%)	4718 (53.1%)	0.36
High CRP risk category, n (%)	3511 (39.5%)	3449 (38.8%)	0.3377
Impaired kidney function, n (%)	737 (8.3%)	722 (8.1%)	0.68

n = Number; SD = standard deviation; SBP = Systolic Blood Pressure; LDL = Low-Density Lipoprotein; HDL = High-Density Lipoprotein; CRP = C-reactive protein.

P-values for categorical variables were determined by likelihood-ratio chi-square tests and for continuous variables by ANOVA F-test.

### Systolic blood pressure

Exploratory analyses found monotonic associations between quartiles of insolation; the lowest, compared to the highest quartile of insolation exposure was associated with 3.5 and 2.3 mmHg higher SBP for unadjusted and fully adjusted models, respectively (Table 2). Confirmatory models replicated this association in the unadjusted model, but the adjusted confirmatory model was not significant. In exploratory analyses insolation significantly interacted with race (Table 3); blacks had stronger associations in both unadjusted and adjusted exploratory models. Blacks, compared to whites, also had a stronger association between insolation and SBP in the confirmatory unadjusted model, but not in the confirmatory adjusted model. After removing temperatures from the fully adjusted confirmatory model, insolation was significant and parameter estimates had similar magnitudes to the unadjusted confirmatory model. Insolation exposure <5^th^ versus ≥5^th^ percentile was significantly associated with 2.4 and 2.5 mmHg higher SBP in exploratory unadjusted and adjusted models, respectively. However, these associations were not significant in confirmatory analyses.

**Table 2:** Differences in continuous secondary cardiovascular endpoints associated with residential insolation exposure in exploratory and confirmatory analyses

**Insolation exposure**	**SBP (mmHg)**	**Total cholesterol (mg/dL)**	**LDL (mg/dL)**	**HDL (mg/dL)**	**LnCRP (mg/dL)**
Quartiles of insolation exposure					
Exploratory analyses – Unadjusted					
1^st^ vs 4^th^ quartile	**3.5 (2.6, 4.5)**	1.5 (−0.7, 3.8)	2.4 (0.5, 4.4)	**−1.3 (−2.2, −0.3)**	**0.0 (−0.1, 0.0)**
2^nd^ vs 4^th^ quartile	**2.3 (1.4, 3.3)**	0.3 (−1.9, 2.6)	0.8 (−1.2, 2.8)	**−2.0 (−2.9, −1.0)**	**0.1 (0.0, 0.1)**
3^rd^ vs 4^th^ quartile	**1.9 (0.9, 2.9)**	0.2 (−2.0, 2.5)	0.4 (−1.6, 2.3)	**−0.9 (−1.8, 0.1)**	**0.1 (0.0, 0.2)**
Exploratory analyses – Fully adjusted					
1^st^ vs 4^th^ quartile	**2.3 (0.8, 3.8)**	−3.1 (−6.6, 0.4)	−1.1 (−4.2, 2.0)	**−2.7 (−4.2, −1.2)**	0.0 (−0.1, 0.1)
2^nd^ vs 4^th^ quartile	**1.9 (0.8, 3.1)**	−2.1 (−4.8, 0.7)	−0.7 (−3.1, 1.7)	**−2.0 (−3.2, −0.9)**	0.1 (0.0, 0.2)
3^rd^ vs 4^th^ quartile	**1.4 (0.4, 2.4)**	−0.8 (−3.1, 1.5)	−0.5 (−2.6, 1.6)	**−0.6 (−1.6, 0.3)**	0.1 (0.0, 0.1)
Confirmatory analyses – Unadjusted					
1^st^ vs 4^th^ quartile	**3.3 (2.3, 4.2)**	N/A	N/A	**−0.2 (−1.1, 0.8)**	**−0.1 (−0.1, 0.0)**
2^nd^ vs 4^th^ quartile	**1.7 (0.8, 2.6)**	N/A	N/A	**−1.8 (−2.8, −0.9)**	**0.0 (−0.1, 0.1)**
3^rd^ vs 4^th^ quartile	**1.8 (0.9, 2.6)**	N/A	N/A	**−0.6 (−1.6, 0.3)**	**0.1 (0.0, 0.2)**
Confirmatory analyses – Fully adjusted					
1^st^ vs 4^th^ quartile	1.6 (−0.5, 3.7)	N/A	N/A	**−1.5 (−3.0, −0.1)**	0.0 (−0.1, 0.1)
2^nd^ vs 4^th^ quartile	1.1 (−0.5, 2.7)	N/A	N/A	**−2.1 (−3.2, −0.9)**	0.0 (−0.1, 0.1)
3^rd^ vs 4^th^ quartile	1.4 (−1.0, 1.7)	N/A	N/A	**−0.5 (−1.4, 0.6)**	0.0 (0.0, 0.1)
Low insolation exposure					
<5^th^ vs ≥5^th^ percentile					
Exploratory analyses - Unadjusted	**2.4 (0.8, 3.9)**	0.5 (−3.1, 4.1)	1.3 (−2.0, 4.5)	−1.0 (−2.6, 0.5)	**−0.2 (−0.3, −0.1)**
Exploratory analyses – Fully adjusted	**2.5 (1.0, 4.1)**	−0.4 (−4.1, 3.3)	0.9 (−2.4, 4.2)	**−1.7 (−3.2, −0.1)**	−0.1 (−0.2, 0.0)
Confirmatory analyses - Unadjusted	1.1 (−0.4, 2.7)	N/A	N/A	−0.1 (−1.6, 1.5)	**−0.1 (−0.2, 0.0)**
Confirmatory analyses – Fully adjusted	0.6 (−1.7, 2.9)	N/A	N/A	0.0 (−1.7, 1.6)	−0.1 (−0.2, 0.0)
High insolation exposure					
<95^th^ vs ≥95^th^ percentile					
Exploratory analyses - Unadjusted	0.3 (−1.3, 1.9)	−1.9 (−5.5, 1.8)	0.4 (−2.8, 3.6)	**−3.6 (−5.1, −2.0)**	0.0 (−0.1, 0.1)
Exploratory analyses – Fully adjusted	0.2 (−1.4, 1.7)	−2.7 (−6.3, 1.0)	0.0 (−3.2, 3.2)	**−2.7 (−4.2, −1.2)**	−0.1 (−0.2, 0.0)
Confirmatory analyses - Unadjusted	N/A	N/A	N/A	**−1.7 (−3.3, −0.2)**	N/A
Confirmatory analyses – Fully adjusted	N/A	N/A	N/A	−1.5 (−3.1, 0.0)	N/A

SBP = Systolic Blood Pressure; LDL = Low-Density Lipoprotein; HDL = High-Density Lipoprotein; CRP = C-reactive protein.

Numbers in parentheses are 95% confidence intervals. Numbers in **bold** are significant (p < 0.05).

All fully adjusted models are adjusted for temperature, age, race, region, gender, education, income, vitamin D intake, alcohol use, smoking, and body mass index. SBP models are further adjusted for antihypertensive medication use. Total cholesterol, LDL, and HDL models are further adjusted for statin use. Models with insolation categorized into quartiles, and using cutoffs at the 5^th^ and 95^th^ percentiles were adjusted for temperature exposures into quartiles and using cutoffs at the 5^th^ and 95^th^ percentiles, respectively.

**Table 3:** Differences in systolic blood pressure (SBP) associated with residential insolation exposure in exploratory and confirmatory analyses, by race

**Quartiles of insolation exposure**	**SBP (mmHg)**		
	**Blacks**	**Whites**	**p-value**
Exploratory analyses – Unadjusted			
1^st^ vs 4^th^ quartile	**4.7 (3.2, 6.1)**	**2.4 (1.1, 3.6)**	
2^nd^ vs 4^th^ quartile	**1.4 (−0.1, 3.0)**	**3.1 (1.9, 4.3)**	**0.0005**
3^rd^ vs 4^th^ quartile	**1.3 (−0.2, 2.8)**	**2.1 (0.9, 3.3)**	
Exploratory analyses – Fully adjusted			
1^st^ vs 4^th^ quartile	**3.8 (2.8, 5.6)**	**1.4 (−0.2, 3.1)**	
2^nd^ vs 4^th^ quartile	**1.1 (−0.5, 2.7)**	**2.4 (1.0, 3.8)**	**0.0012**
3^rd^ vs 4^th^ quartile	**1.2 (−0.2, 2.7)**	**1.5 (0.2, 2.7)**	
Confirmatory analyses – Unadjusted			
1^st^ vs 4^th^ quartile	**4.6 (3.2, 6.1)**	**1.9 (0.7, 3.1)**	
2^nd^ vs 4^th^ quartile	**1.8 (0.2, 3.3)**	**1.9 (0.7, 3.0)**	**0.0058**
3^rd^ vs 4^th^ quartile	**1.7 (0.2, 3.2)**	**1.7 (0.5, 2.9)**	
Confirmatory analyses – Fully adjusted			
1^st^ vs 4^th^ quartile	1.7 (−0.7, 4.1)	1.6 (−0.9, 4.1)	
2^nd^ vs 4^th^ quartile	0.6 (−1.5, 2.6)	1.6 (−0.4, 3.7)	0.5855
3^rd^ vs 4^th^ quartile	−0.3 (−2.3, 1.6)	1.0 (−0.8, 2.8)	

Numbers in parentheses are 95% confidence intervals. Numbers in **bold** are significant (p < 0.05).

All fully adjusted models are adjusted for temperature, age, race, region, gender, education, income, vitamin D intake, alcohol use, smoking, body mass index, and antihypertensive medication use. Models with insolation categorized into quartiles, and using cutoffs at the 5^th^ and 95^th^ percentiles were adjusted for temperature exposures into quartiles and using cutoffs at the 5^th^ and 95^th^ percentiles, respectively.

### Total cholesterol, LDL, and HDL

Insolation did not have significant associations with total cholesterol or LDL in exploratory models (Table 2). Lower quartiles of insolation exposure were significantly associated with lower HDL levels in all analyses. The lowest, compared to the highest quartile of insolation exposure was significantly associated with 1.3 and 1.5 mg/dL lower HDL levels in adjusted exploratory and confirmatory models, respectively. Insolation exposure <5^th^ versus ≥5^th^ percentile was associated with lower HDL levels in the adjusted exploratory, but not adjusted confirmatory model. Insolation exposure <95^th^ versus ≥95^th^ percentile was associated with 2.7 mg/dL lower HDL levels in the adjusted exploratory model, but this association was not significant in the confirmatory model.

### Continuous C-reactive protein

Quartiles of insolation, and insolation exposure <5^th^ versus ≥5^th^ percentile were each significantly associated with differences in CRP levels in unadjusted exploratory and confirmatory models, but these associations were no longer significant after adjustment. Insolation exposure <95^th^ versus ≥95^th^ percentile was not associated with a difference in CRP levels in any models (Table 2).

### Hypertension

Insolation was not significantly associated with hypertension in any analyses (Table 4).

**Table 4:** Hazard ratios for continuous secondary cardiovascular endpoints associated with residential insolation exposure in exploratory and confirmatory analyses

**Insolation exposure**	**Hypertension**	**Dyslipidemia**	**High CRP**	**Impaired kidney**
Quartiles of insolation exposure				
Exploratory analyses – Unadjusted				
1^st^ vs 4^th^ quartile	1.1 (0.9, 1.2)	1.0 (0.9, 1.1)	**0.9 (0.8, 1.1)**	0.8 (0.7, 1.0)
2^nd^ vs 4^th^ quartile	1.0 (0.9, 1.2)	1.1 (1.0, 1.2)	**1.1 (0.9, 1.2)**	0.9 (0.8, 1.2)
3^rd^ vs 4^th^ quartile	1.2 (1.0 (1.3)	1.0 (0.9, 1.2)	**1.2 (1.0, 1.3)**	0.9 (0.7, 1.1)
Exploratory analyses – Fully adjusted				
1^st^ vs 4^th^ quartile	1.2 (0.9, 1.4)	0.9 (0.8, 1.1)	1.1 (0.9, 1.3)	0.8 (0.6, 1.2)
2^nd^ vs 4^th^ quartile	1.0 (0.9, 1.2)	1.0 (0.9, 1.2)	1.2 (1.0, 1.4)	0.9 (0.7, 1.2)
3^rd^ vs 4^th^ quartile	1.1 (0.9, 1.2)	1.0 (0.9, 1.1)	1.1 (1.0, 1.3)	0.9 (0.7, 1.2)
Confirmatory analyses – Unadjusted				
1^st^ vs 4^th^ quartile	N/A	N/A	1.0 (0.8, 1.1)	N/A
2^nd^ vs 4^th^ quartile	N/A	N/A	1.0 (0.9, 1.1)	N/A
3^rd^ vs 4^th^ quartile	N/A	N/A	1.1 (1.0, 1.2)	N/A
Confirmatory analyses – Fully adjusted				
1^st^ vs 4^th^ quartile	N/A	N/A	1.1 (0.9, 1.4)	N/A
2^nd^ vs 4^th^ quartile	N/A	N/A	1.0 (0.8, 1.2)	N/A
3^rd^ vs 4^th^ quartile	N/A	N/A	1.0 (0.9, 1.2)	N/A
Low insolation exposure				
<5^th^ vs ≥5^th^ percentile				
Exploratory analyses - Unadjusted	1.0 (0.8, 1.2)	1.1 (0.9, 1.3)	**0.8 (0.6, 0.9)**	0.8 (0.6, 1.2)
Exploratory analyses – Fully adjusted	1.2 (1.0, 1.5)	1.2 (1.0, 1.5)	0.9 (0.7, 1.1)	0.8 (0.6, 1.3)
Confirmatory analyses - Unadjusted	N/A	N/A	0.9 (0.7, 1.1)	N/A
Confirmatory analyses – Fully adjusted	N/A	N/A	0.9 (0.7, 1.2)	N/A
High insolation exposure				
<95^th^ vs ≥95^th^ percentile				
Exploratory analyses - Unadjusted	1.0 (0.8, 1.2)	1.2 (1.0, 1.5)	1.0 (0.9, 1.3)	0.8 (0.6, 1.1)
Exploratory analyses – Fully adjusted	0.9 (0.8, 1.2)	1.2 (0.9, 1.4)	0.9 (0.7, 1.1)	0.8 (0.6, 1.2)
Confirmatory analyses - Unadjusted	N/A	N/A	N/A	N/A
Confirmatory analyses – Fully adjusted	N/A	N/A	N/A	N/A

CRP = C-reactive protein.

Numbers in parentheses are 95% confidence intervals. Numbers in **bold** are significant (p < 0.05).

Fully adjusted models are adjusted for temperature, age, race, region, gender, education, income, vitamin D intake, alcohol use, smoking, and body mass index. Models with insolation categorized into quartiles, and using cutoffs at the 5^th^ and 95^th^ percentiles were adjusted for temperature exposures into quartiles and using cutoffs at the 5^th^ and 95^th^ percentiles, respectively.

### Dyslipidemia

Insolation was not significantly associated with dyslipidemia in any main effect models (Table 4). In exploratory models, insolation exposure <5^th^ versus ≥5^th^ percentile was associated with an increased risk of dyslipidemia among those without impaired kidney function, but associated with a decreased risk of dyslipidemia among those without impaired kidney function (Additional file 2: Table S2). However, this was not replicated in confirmatory models.

### High levels of C-reactive protein

Quartiles of insolation exposure were significantly associated with high CRP in the unadjusted exploratory model (Table 4). However, this association was not monotonic. In the unadjusted exploratory, insolation exposure <5^th^ versus ≥5^th^ percentile was significantly associated with high CRP. In unadjusted exploratory models, quartiles of insolation exposure significantly interacted with kidney impairment and insolation exposure <5^th^ vs ≥5^th^ percentile significantly interacted with race (Additional file 3: Tables S3 and Additional file 4: Table S4). However, no insolation main effects or interaction terms in exploratory adjusted models or in any of the confirmatory models were significant.

### Kidney impairment

Insolation was not significantly associated with kidney impairment in any analyses (Table 4).

## Discussion

This analysis adds to the limited previous research addressing the relationship between sunlight and vascular health. Higher myocardial infarction, stroke, and adverse vascular risk factor rates have been reported in farther northern latitudes, but it is not clear whether this is due to environmental, social, or other factors [4,29,30]. There is also some evidence of higher myocardial infarction and stroke rates during the winter [1,31] although other research contradicts this [3]. While lower temperatures have been shown to be associated with high blood pressures [2,32], there may also be seasonal variations in lipid levels that are independent of temperature [33]. Sunlight exposure is another seasonal factor, and might affect vascular risk factors through vitamin D metabolism, which is increasingly found to be related to various chronic diseases. There is indication that vitamin D insufficiency may increase vascular event risk [34,35] and adversely impact various vascular risk factors [8]. For most people, vitamin D status is primarily determined by sunlight exposure [6,36]. Blood serum 25(OH)D levels are usually used to determine vitamin D status and can fluctuate with differential exposure to light and dietary intake.

This study is the third using REGARDS data merged with NASA meteorological data that demonstrated a possible link between sunlight and health [20,27]. The results of this study suggest that lower long-term sunlight exposure has an association with lower HDL levels, after accounting for confounders. Since this association was found in both exploratory and confirmatory models, it is not likely that this finding is due to chance. However, the magnitude of this association is small, since those in the lowest, compared to the highest quartile of insolation exposure had only about 2 mg/dL lower HDL levels compared to those with higher sunlight exposures. In addition, while observational studies have shown that higher HDL levels are associated with lower cardiovascular risk, interventional studies have not been consistent and are ongoing, so the clinical significance of this association is unknown [37]. Sunlight also had significant univariate relationships with SBP in both exploratory and confirmatory models, but this association did not remain significant after confounder adjustment in the confirmatory models. In addition, the associations with SBP were also small, with adjusted effect sizes less than 3 mmHg. We also found that the association between insolation and SBP may be stronger among blacks than whites, but this interaction was also not significant in adjusted confirmatory models. We determined that this was due to the inclusion of temperatures in the model. Previous research in REGARDS and other studies have found lower temperatures to be related to higher blood pressures [38]. It is not clear why the inclusion of temperature would eliminate the significant association between insolation and SBP in the exploratory, but not confirmatory analyses. Collinearity may be an issue, with higher maximum temperatures correlated with higher insolation levels. These results agree with our previous analyses which suggest that decreased sunlight exposure is related to increased stroke incidence and increased likelihood of cognitive impairment and decline [20,27,39], although given the effect sizes it is unlikely that the traditional risk factors explored fully mediate these associations.

There is little other research examining sunlight and vascular risk, but a recent Hong Kong study found that temperature and air pressure, but not solar radiation, were significantly associated with stroke [40]. Our current and previous research differs from this study in many ways, including temporality (exposure within days of outcome, rather than one year exposure in this analysis), location (a small tropical area rather than a wide range of mostly temperate areas), and that our study did not include air pressure. Since 25(OH)D3 has a biological half-life of several weeks [34], it would be plausible that our longer term sunlight exposure would have a larger effect on vitamin D levels, and thus on vascular risk. And while we did not include air pressure in our models, we did account for temperature, as this is the primary meteorological variable that has been shown to have associations with vascular risk.

Exposure misclassification exists as a possible source of bias. This could happen if during the time period of an exposure measurement a participant spent a large amount of time in a climate different from that indicated by the outdoor exposures linked to his or her residence. In addition to exposure misclassification, it is possible that our findings are confounded by spatial autocorrelation, although that is not likely since adding region to the model did not attenuate the relationship. Another potential limitation is that there may be confounders for which we have not accounted, such as air pollution. While we did not correct for variables such as cloudiness and altitude, insolation measures represent the sunlight energy received on the ground, so the effects of such variables do not need to be included.

## Conclusions

This study found a relationship between sunlight exposure and HDL levels that is not likely due to chance. However, all observed associations were of small magnitudes. More research is needed to determine whether insolation affects cardiovascular outcomes through these risk factors.

## Abbreviations

CRP: C-reactive protein; DBP: Diastolic blood pressure; ECG: Electrocardiogram; eGFR: Estimated glomerular filtration rate; HDL: High-density lipoprotein; LDL: High-density lipoprotein; NOAA: National Oceanic and Atmospheric Administration; NLDAS-2: North American Land Data Assimilation System Phase 2; REGARDS: REasons for Geographic and Racial Differences; SBP: Systolic blood pressure.

## Competing interests

The authors declare that they have no competing interest.

## Authors’ contributions

STK helped originate the hypothesis, managed the data, performed the analyses, and drafted the manuscript. MC provided medical, physiological, and epidemiological expertise. GH provided epidemiological expertise and contributed to the study design. SEJ provided physiological and epidemiological expertise. WLC managed the data and provided meteorological expertise. MZA managed the data. LAM helped originate the manuscript hypothesis, provided statistical expertise, and contributed to the study design. All authors helped to edit the manuscript, and have read and approved the final manuscript.

## Pre-publication history

The pre-publication history for this paper can be accessed here:

http://www.biomedcentral.com/1471-2377/14/133/prepub

## Supplementary Material

Additional file 1: Table S1 Confounder distributions in exploration (n = 8886) and confirmatory (n = 8887) samples; Provides a table of REGARDS participant baseline characteristics in the exploratory and confirmatory samples. Click here for file

Additional file 2: Table S2 Hazard ratios (95% confidence intervals) for dyslipidemia associated with residential insolation exposure in exploratory and confirmatory analyses, by kidney impairment; Provides a table examining the interaction between low insolation exposure and kidney impairment on dyslipidemia. Click here for file

Additional file 3: Table S3 Hazard ratios (95% confidence intervals) for high C-reactive protein associated with residential insolation exposure in exploratory and confirmatory analyses, by kidney impairment; Provides a table examining the interaction between quartiles of insolation exposure and kidney impairment on high CRP. Click here for file

Additional file 4: Table S4 Hazard ratios (95% confidence intervals) for high C-reactive protein associated with residential insolation exposure in exploratory and confirmatory analyses, by race; Provides a table examining the interaction between low insolation exposure and race on high CRP. Click here for file

## Acknolwedgements

The authors thank the other investigators, the staff, and the participants of the REGARDS study for their valuable contributions. A full list of participating REGARDS investigators and institutions can be found at http://www.regardsstudy.org.

The NLDAS hourly data used in this study were acquired as part of the mission of NASA’s Earth Science Division and archived and distributed by the Goddard Earth Sciences (GES) Data and Information Services Center (DISC).

This work was supported by the National Institute of Neurological Disorders and Stoke [U01 NS041588]; and the National Aeronautics and Space Administration [grant# NNX09AV81G].
